# Engineered PUF proteins: new flexible toolkits to target the replication of RNA viruses

**DOI:** 10.2217/fvl-2020-0134

**Published:** 2021-01-14

**Authors:** Seyed Jalal Kiani, Zohreh Yousefi Ghalejoogh, Katayoun Samimi-Rad

**Affiliations:** 1 ^1^Department of Virology, School of Medicine, Iran University of Medical Sciences, Tehran, Iran; 2 ^2^Department of Virology, School of Public Health, Tehran University of Medical Sciences, Tehran, Iran

**Keywords:** hepatitis C virus, Huh7.5 cells, PUF proteins, RNA-binding proteins

## Abstract

**Aim:** The RNA recognition code of an RNA-binding protein known as Pumilio/FBF (PUF) protein was reprogrammed in order to provide binding to internal ribosome entry site (IRES) of hepatitis C virus (HCV) genome. **Materials & methods:** The ability of the modified protein to repress IRES-dependent translation was analyzed by dual-luciferase reporter assay, cell viability assay, cell cytotoxicity assay and anti-HCV assay. **Results:** The modified protein was able to reduce reporter gene expression (>30%) and HCV viral load (>98%) and reduced HCV-induced cytotoxicity to the level observed in uninfected cells. **Conclusion:** Our results can set the stage for using modified PUFs for interfering with critical steps such as replication and translation in virus life cycle, especially RNA viruses.

Interactions between viruses and hosts often lead to different types of outcomes and complications in infected hosts. Therefore, investigations for new approaches that inhibit virus replication cycle have been continued unabated. Among possible approaches, the application of nucleic acid-binding proteins is an attractive path to explore. In previous studies, RNA-binding proteins (RBPs) that repress RNA replication of plant viruses by directly binding to the viral RNA have been described as unique defense mechanisms against these viruses [1,2].

Among several families of RBPs, Pumilio/FBF (PUF) proteins function as post-transcriptional and translational regulators by interacting with sequence-specific motifs in the 3′-untranslated region (UTR) of their target mRNAs. They contain a highly conserved RNA-binding domain, known as the Pumilio-homology domain (PUM-HD). This domain with eight tandem repeats can recognize eight RNA bases (the consensus 5′-UGUAUAUA-3′ in the case of *Homo sapiens* PUM-HD), in an antiparallel mode, with a relatively simple code. Interestingly, this recognition code can be reprogrammed in order to change the sequence-specificity of the PUM-HD for binding to the sequence of interest [3–5].

RNA viruses, in particular viruses with a single-stranded genomic RNA such as hepatitis C virus (HCV), are ideal candidates for investigating the inhibitory effect of RBPs that interact with specific motifs in their RNA target. HCV 5′-UTR contains a structured RNA element with three domains called the internal ribosome entry site (IRES). Domain III of the HCV IRES, which has the highest degree of conservation, plays an important role in initiation of the viral polyprotein translation and is required for high-level RNA replication [6,7].

In our previous study, sequence-specific binding of a modified PUM-HD protein to HCV IRES was assessed by a RNA-protein pull-down assay as well as a dual-luciferase reporter assay in HEK-293 cells [8]. In this study, the efficiency of the modified PUM-HD protein to repress virus replication was investigated in human hepatoma cell line (Huh7.5) infected with genotype 2a HCV JFH-1 strain. By using this protein, we showed successful reduction of IRES-dependent translation and virus replication in Huh7.5 cell culture.

## Materials & methods

### Basic strategy

In our strategy, the sequence-specific RNA-binding domain of *Homo sapiens* Pumilio1 (HsPUM1-HD) was used. This domain was modified to bind to an 8-nucleotide sequence (5′-UGGAUAAA-3′) of the stem-loop IIIb region in HCV IRES. This region is one of the most accessible sites in the HCV 5′-UTR and is involved in recruitment of eIF-3 and 40S ribosomal subunit [9]. In order to make the wild-type HsPUM1-HD (wtPUM) capable to recognize specifically our target sequence of HCV IRES, the following modifications were applied in modified HsPUM1-HD (mPUM) sequence as indicated in Table 1. Briefly, to switch the recognition code of the repeat 6 (R6) from U to G, Gln-1047, Tyr-1044 and Asn-1043 were substituted by glutamic acid, asparagine and serine, respectively. In addition, to switch the recognition code of the repeat 2 (R2) from U to A, Gln-903, Tyr-900 and Asn-899 were substituted by glutamine, asparagine and cysteine, respectively.

**Table 1.** Engineered modifications in HsPUM1-HD sequence. The wild-type HsPUM1-HD sequence was modified to recognize RNA sequence of HCV IRES stem-loop IIIb (5′-UGGAUAAA-3′) instead of the consensus recognition sequence of 5′-UGUAUAUA-3′.

PUM-HD repeat	R8	R7	R6	R5	R4	R3	R2	R1
**Wild-type HsPUM1-HD (wtPUM)**	Q-1126	E-1083	Q-1047	Q-1031	Q-975	Q-939	Q-903	Q-867
	Y-1123	N-1080	Y-1044	R-1008	H-972	R-936	Y-900	R-864
	N-1122	S-1079	N-1043	C-1007	N-971	C-935	N-899	S-863
Consensus RNA sequence	5′-U	G	U	A	U/C	A	U	A-3′
PUM-HD repeat	R8	R7	**R6**	R5	R4	R3	**R2**	R1
**Modified HsPUM1-HD (mPUM)**	Q-1126	E-1083	**E-1047**	Q-1031	Q-975	Q-939	**Q-903**	Q-867
	Y-1123	N-1080	**N-1044**	R-1008	H-972	R-936	**N-900**	R-864
	N-1122	S-1079	**S-1043**	C-1007	N-971	C-935	**C-899**	S-863
HCV IRES sequence	5′-U	G	**G**	A	U	A	**A**	A-3′

Bold text shows the modifications applied in wtPUM sequence.

### Dual luciferase reporter assay

Human embryonic kidney (HEK) 293T cells were cultured in Dulbecco’s modified Eagle’s medium (DMEM) (Gibco, NY, USA) supplemented with 10% heat-inactivated fetal calf serum (FCS) and antibiotics (100 IU of penicillin and 100 μg/ml streptomycin) at 37°C under 0.5% CO_2_. The cells were seeded in 96-well plates at a density of 2 × 10^4^ cells per well, 24 h before transfection. The cells were then cotransfected with psiCHECK2-IRES (40 ng/μl) and either pcDNA-wtPUM or pcDNA-mPUM plasmid (400 ng/μl) using FuGENE^®^6 transfection reagent (Promega Corporation, WI, USA), according to the manufacturer’s protocol. Dual luciferase reporter assay kit (Promega) was used according to the manufacturer’s instruction in order to analyze the expression level of both luciferase enzymes 72 h post-transfection. Three independent experiments were performed in at least triplicates and the data were shown as mean ± standard deviation (SD).

### Viability assay in Huh7.5 clone 5 cells

Viability assay was performed using CytoTox 96^®^ Non-Radioactive Cytotoxicity Assay (Promega), with some modifications to the manufacturer’s instruction. This colorimetric assay quantitates lactate dehydrogenase (LDH), an enzyme that is stably produced in cytosol and released upon cell lysis. Here, the kit was used to evaluate the amount of LDH release from all lysed cells in medium, which represents the number of viable cells. Briefly, Huh7.5 clone 5 cells were seeded in 96-well plates (10^4^ cells per well) and transfected with either pcDNA-wtPUM or pcDNA-mPUM plasmid for 72 h. Three independent experiments were performed in at least triplicates and the data were shown as mean ± SD.

### Anti-HCV assay

To determine whether mPUM is able to reduce HCV replication, Huh7.5 cells were first cultured in DMEM-GlutaMAX™ (Gibco) supplemented with 10% heat-inactivated FCS and antibiotics (100 IU of penicillin and 100 μg streptomycin per ml) in a 0.5% CO_2_ enriched atmosphere at 37°C. Huh7.5 cells were then seeded in T75 flasks at a density of 4 × 10^6^ cells per flask, and transiently transfected 24 h later by 3 μg of wtPUM- or mPUM-expressing plasmids using FuGENE^®^6 transfection reagent (Promega), according to the manufacturer’s protocol. 48 h after transfection, the cells were infected by incubation for 12 h with 2 × 10^5^ focus-forming unit (FFU) of the JFH-1 strain that had been optimized by ten cycles of infection in naive Huh7.5 cells [10]. The replication-deficient JFH-1 GND with a point mutation in GDD motif of the NS5B was used as a negative control. Total RNA was isolated from the infected cell pellets on days 3, 6 and 9 postinfection using Nucleospin RNA^®^ kit (Macherey Nagel, Düren, Germany). The isolated RNA (500 ng) was used for cDNA synthesis using Superscript™ First-Strand II Synthesis System for real-time (RT)-PCR (Fisher Scientific, Loughborough, UK). Viral RNA was then quantified by quantitative RT-PCR using the LightCycler^®^ 480 SYBRGreen I Master mix (Roche, Basel, Switzerland) and primers-specific JGH-1 virus (5′-UTRf: 5′-CgCTCAATgCCTggAgATTTg-3′; 5′-UTRr: 5′-gCACggTCTACgAgACCTCC-3′). RNA integrity and cDNA synthesis were checked by amplifying the β-actin cDNA (Actinf: 5′-CgCACCACTggCATTgTCAT-3′; Actinr: 5′-TTCTCCTTgATgTCACgCAC-3′). The results of two independent experiments were performed in quadruplicate are shown, expressed in absolute numbers of genome copies per ml.

### Cell cytotoxicity assay

In order to further analyze the effect of mPUM protein on virus replication, cell cytotoxicity assay was performed on cells transfected with the plasmids of interest (pcDNA-wtPUM or pcDNA-mPUM) and infected with the JFH-1 virus. The CytoTox 96^®^ Non-Radioactive Cytotoxicity Assay (Promega) was used to evaluate the cell cytotoxicity, according to the manufacturer’s instructions. Briefly, Huh7.5 cells of interest collected at day 3, 6 and 9 postinfection were seeded in 96-well plates (15,000 cells per well; six wells per condition) and naive Huh7.5 cells and medium were used as controls. Twenty hours later, cells of interest (three out of six wells) were lysed by adding 10 μl of lysis 10X solution to determinate the maximum LDH release. After 45 min of incubation at 37°C, supernatants (50 μl of each six wells) were transferred to a fresh 96-well plate, reconstituted substrate mix (50 μl) was added to each well and the enzymatic reaction was allowed to proceed for 30 min at room temperature. The enzymatic assay was then stopped by adding 50 μl/well of the stop solution and absorbance at 490 nm was recorded using an ELISA plate reader. The cytotoxicity percent was determined using this formula:

$$\%\;\text{Cytoxicity}=\frac{\text{Experimental LDH release }(\text{OD}490)}{\text{Maximum LDH release }(\text{OD}490)}$$

### Western blot

Total protein was extracted from Huh7.5 clone 5 cells, using lysis buffer (1% Triton-X100, and 1% protease inhibitors, including phenylmethylsulfonyl fluoride (PMSF), aprotenin and leopeptin in phosphate-buffered saline [PBS]). In brief, cell pellets were resuspended in lysis buffer, incubated on ice for 10 min, centrifuged at top speed at 4°C for 10 min and the supernatant was stored at -80°C. Protein concentration was quantified by DS-11 spectrophotometer (DeNovix, DE, USA) and total protein for each sample was normalized and separated on 12% SDS-polyacrylamide gel electrophoresis. Beta-actin was used as the housekeeping gene control. Protein transfer to nitrocellulose membrane (Amersham Pharmacia Biotech, Freiburg, Germany) was performed at 100 V for 60 min and the membrane was then blocked with 2% milk protein in tris-buffered saline (TBS) buffer for 60 min at room temperature (RT). Then, the membrane was incubated with mouse anti-NS3, anti-His tag (wtPUM and mPUM) or anti-β actin primary antibodies (1:1000 dilution; Abcam, MA, USA) at 4°C, overnight. After four washes with tris-buffered saline with Tween 20 (TBST) for 10 min at RT, the membrane was incubated with goat anti-mouse horseradish peroxidase (HRP)-conjugated secondary antibody (1:2000 dilution; Abcam) for 60 min at RT. The membrane was washed again and protein bands were visualized using enhanced chemiluminescence (ECL) kit (Amersham) according to the manufacturer’s instruction.

### Statistical analysis

Statistical analyses were performed using GraphPad Prism 6 software and analysis of variance (one-way or two-way ANOVA) was applied to compare differences between means. A result was considered statistically significant if the p-value < 0.05 (*p < 0.05; **p < 0.01; ***p < 0.001; ****p < 0.0001).

## Results

### Modified PUM reduced IRES-dependent luciferase translation

**Figure 1.**
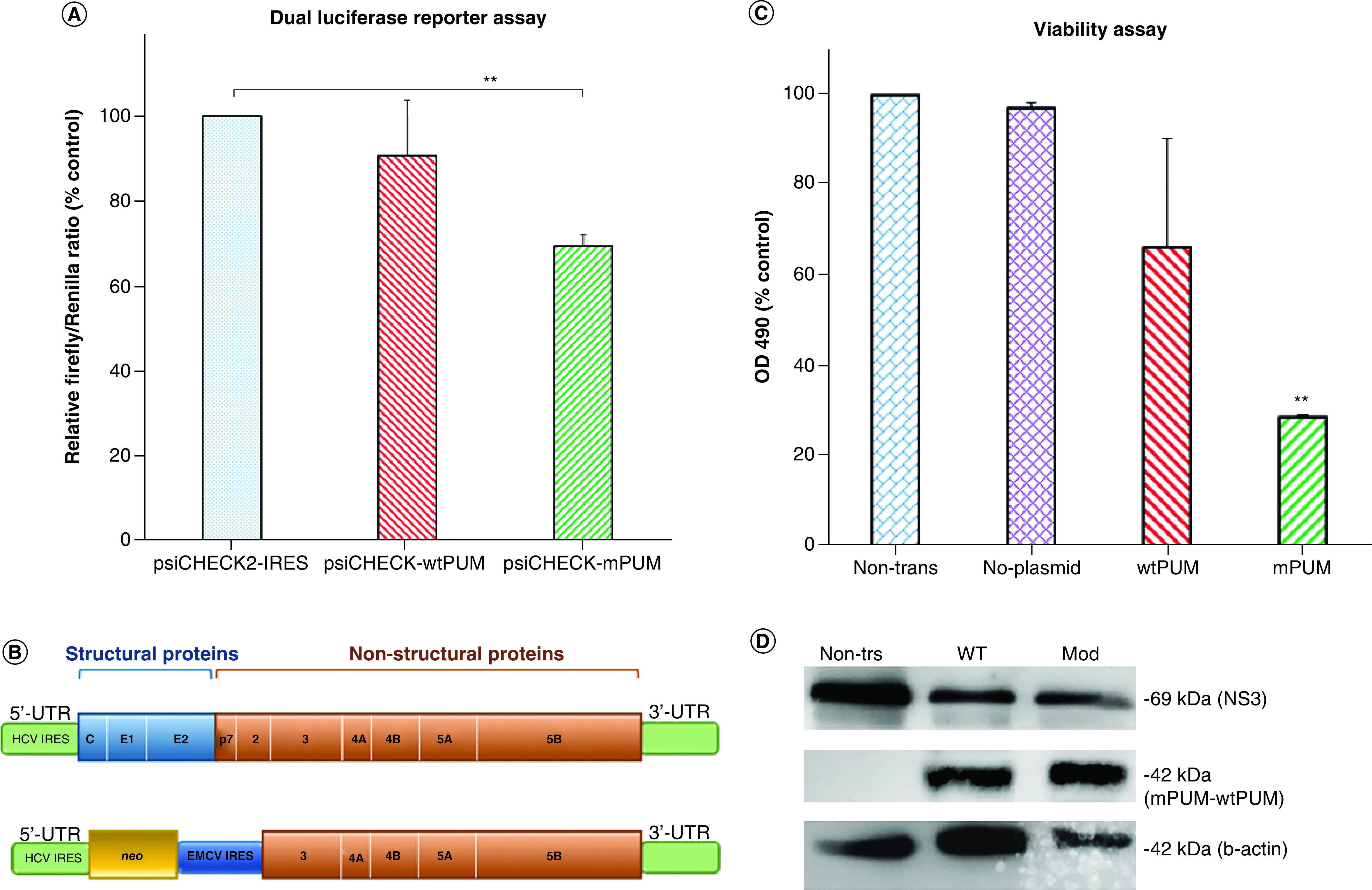
The effect of mPUM protein on internal ribosome entry site-dependent translation. **(A)** Dual luciferase reporter assay in HEK 293T cells. The cells were transfected by a plasmid-expressing Firefly luciferase under the control of HCV IRES (psiCHECK2-IRES) as control. Moreover, the cells were cotransfected with psiCHECK2-IRES and either a plasmid-expressing wtPUM or mPUM protein. Firefly luciferase expression level was evaluated 72 h after cotransfection. **(B)** Schematic organization of HCV genome (top) and a subgenomic replicon (bottom). While the translation of the genome to the viral polyprotein is HCV IRES-dependent in the bicistronic replicon system, translation of nonstructural proteins is EMCV IRES-dependent and translation of *neo* gene is HCV IRES-dependent. **(C)** Cell viability assay in Huh7.5 clone 5 cells. The effect of mPUM expression on the viability of cells expressing neo-resistant gene under the control of HCV IRES was evaluated at 72 h post-transfection in the presence of G-418 antibiotic selection pressure. **(D)** Western blot analysis for viral NS3 as well as wtPUM and mPUM expression. Cell lysates of naive nontransfected, wtPUM- and mPUM-transfected Huh7.5 clone 5 cells were analyzed. Beta-actin was used as a loading control (**p < 0.01). EMCV: Encephalomyocarditis virus; HCV: Hepatitis C virus; IRES: Internal ribosome entry site.

In order to check the effect of mPUM protein on IRES-dependent translation, a dual luciferase reporter assay was performed. For this, the psiCHECK2 plasmid (Promega Corporation) was modified to harbor HCV IRES between its two luciferase genes, as described previously [8]. Transcription from this modified plasmid (psiCHECK2-IRES) results in a bicistronic mRNA, in which the translation of Renilla luciferase is cap-dependent, while the translation of Firefly luciferase is IRES-dependent. Our results showed that the expression of mPUM protein in HEK 293T cells expressing the bicistronic psiCHECK2-IRES mRNA resulted in about 31% (p = 0.0027) decrease in Firefly expression in comparison with the cells expressing only psiCHECK2-IRES mRNA (Figure 1A). Although the expression of wtPUM also induced an approximate 10% reduction in Firefly expression compared with the cells expressing only psiCHECK2-IRES mRNA, however, this reduction was not statistically significant (p = 0.5644).

### Modified PUM induced repression of IRES-dependent *neo* translation

In order to further check the ability of mPUM protein in binding to HCV IRES and suppression of its role in translation, Huh7.5 clone 5 cells containing autonomously replicating HCV RNAs (replicons) were cultured in DMEM (Gibco) supplemented with 10% heat-inactivated FCS and antibiotics (100 IU of penicillin, 100 μg/ml streptomycin and 800 μg/ml geneticin) at 37°C under 0.5% CO_2_. The replicons are subgenomic constructs that can replicate autonomously and express viral nonstructural proteins, including RNA-dependent RNA polymerase. They contain the exact sequence of HCV 5′- and 3′-UTRs [11]. There is a neomycin phosphotransferase gene (*neo*) just downstream of the IRES of the virus to ensure cell resistance to neomycin and its analogs such as Geneticin (G-418). The encephalomyocarditis virus IRES is also recruited downstream of the *neo* gene and just upstream of the viral nonstructural genes to ensure the translation of several subunits of the HCV replicase complex (Figure 1B). Our hypothesis was that the binding of mPUM to HCV IRES is able to repress neomycin phosphotransferase gene translation and this will increase cell cytotoxicity in the presence of G-418. As demonstrated in Figure 1C, our results showed that the cell viability of clone 5 cells decreased to 28.41% (p: 0.0036) at 72 h after transfection. Although cell viability decreased also in cells transfected with wtPUM, analyses showed that this reduction was not statistically significant (p: 0.2971). The expression of wtPUM and mPUM proteins as well as viral NS3 protein was assessed by western blot analysis (Figure 1D).

### Modified PUM reduced HCV replication

**Figure 2.**
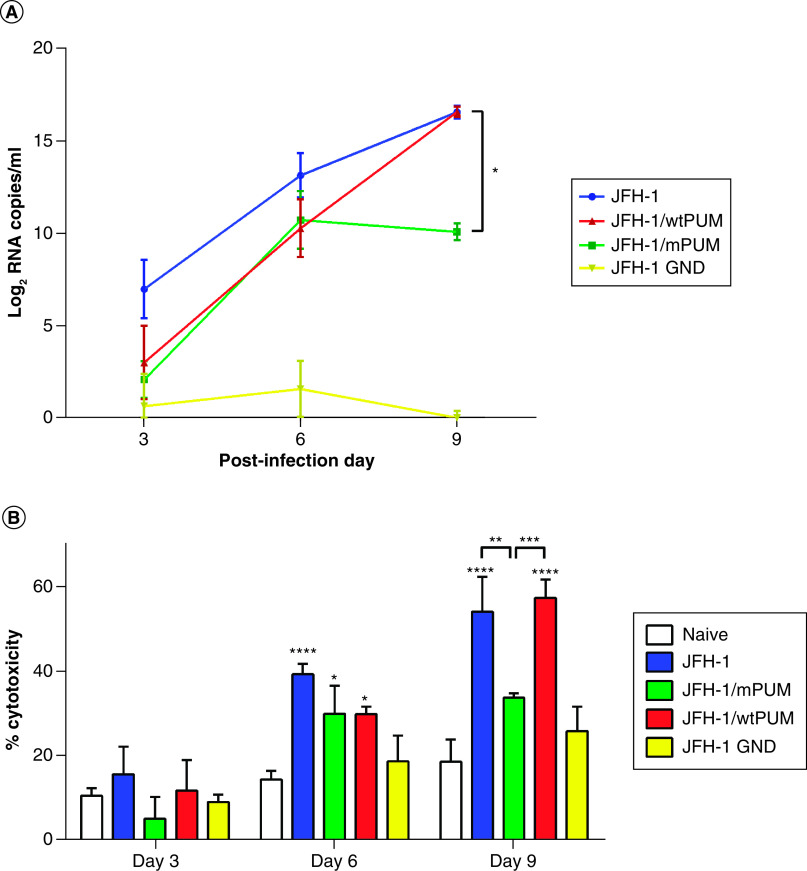
The effect of mPUM protein on hepatitis C virus replication. **(A)** Impact of mPUM protein expression on HCV viral load. Huh7.5 cells were transfected with pcDNA-mPUM or pcDNA-wtPUM, and then infected 48 h later with the JFH-1 virus. The JFH-1 GND strain infection and naive Huh7.5 cells were used as controls. Quantitative RT-PCR assay was performed on days 3, 6 and 9 after the infection. All data were normalized by naive Huh7.5 rates and one-way ANOVA test was used to determine statistical significance. The results are expressed as absolute numbers of genome copies per ml (mean ± SEM, n = 2). **(B)** Impact of mPUM protein expression on JFH1-induced cytopathic effects in Huh7.5 cells. Cytotoxicity assay was performed on mPUM- and wtPUM-expressing JFH1-infected Huh7.5 cells as well as JFH1-infected cells on days 3, 6 and 9 postinfection. The JFH-1 GND strain infection and naive Huh7.5 cells were used as controls. One-way ANOVA test was used to compare the results, which are expressed as mean percentage of cytotoxicity (mean ± SEM, n = 2). p-values less than 0.05 were considered significant. The asterisk symbol (*) above each bar is in comparison with naive Huh7.5 cells. ANOVA: Analysis of variance; HCV: Hepatitis C virus; RT-PCR: Real-time PCR; SEM: Standard error of the mean.

To determine whether mPUM is able to reduce HCV replication, Huh7.5 cells were transfected with wtPUM- or mPUM-expressing plasmids, 24 or 48 h before infection with JFH-1 virus. As shown in Figure 2A, the expression of neither mPUM nor wtPUM proteins caused significant decrease in viral RNA copies/ml, on days 3 and 6. However, mPUM expression for 48 h before infection significantly induced a more than 98% decrease (p: 0.017) in JFH-1 viral load on day 9 when compared with both control JFH-1 infected and wtPUM-expressing cells (Figure 2A). The viral load of JFH-1 GND strain was under the detection limit.

### Modified PUM reduced cell cytotoxicity of HCV-infected cells

In order to further analyze the effect of mPUM protein on virus replication, cell cytotoxicity assay was performed on JFH-1-infected cells. Our cytotoxicity assay showed that on day 3, there was no significant difference between infected and naive Huh7.5 cells (Figure 2B). This indicates that JFH-1 replication has no obvious cytopathic effect (CPE) on Huh7.5 cells up to day 3.

Comparison of cell cytotoxicity between infected and naive cells on day 6 and also on day 9 showed a statistically significant increase in cytotoxicity for infected cells in comparison to naive cells. This elucidates that the CPE of the JFH-1 infection started from day 6 with this optimized strain. Cell viability was also significantly impacted on day 6 for cells transfected with pcDNA-wtPUM and pcDNA-mPUM plasmids and infected with JFH-1 virus. However, JFH-1 infected cells showed no significant difference in cytotoxicity values on day 6 when compared with the cells transfected with pcDNA-wtPUM and pcDNA-mPUM plasmids and infected with JFH-1 virus. Finding no significant difference between the cells transfected with wtPUM or mPUM expressing plasmids and infected with JFH-1 virus supports the lack of cytotoxicity induction by the engineered modifications in HsPUM1-HD.

Interestingly, the expression of the modified protein in the infected cells reduced cytotoxicity close to the level of naive cells on day 9 (Figure 2B). This decline was specific to the mPUM since it was not observed in JFH-1-infected cells expressing wtPUM. Altogether, these results demonstrated that mPUM protein is able to enhance cell viability of JFH-1 infected cells to the level of naive cells, most likely through the repression of virus replication.

## Discussion

This study is the first report of a successful repression of an RNA virus replication by an engineered sequence-specific RBP in cell culture. Although the precise mechanism of the mPUM protein involvement in the reduction of HCV replication needs further investigation, it is most likely that this reduction is due to the binding to HCV IRES stem-loop IIIb. As a part of IRES structure, stem-loop IIIb plays a critical role in translation of the viral polyprotein through interaction with eIF-3 and 40S ribosomal subunit [9]. Any obstacle to such an interaction can decrease viral polyprotein translation. This is in agreement with our previous results of a luciferase reporter assay that showed more than 30% decrease in IRES-dependent translation of Firefly luciferase in the presence of mPUM protein targeted for stem-loop IIIb [8]. This was also in line with the results of our viability assay in Huh7.5 clone 5 cells, where the expression of mPUM protein in clone 5 cells in a medium supplemented with G-418 resulted in more than 70% reduction in cell viability. Although further studies are required, the potential binding of mPUM protein to HCV IRES in the replicon RNA may suppress IRES-dependent translation of neomycin phosphotransferase gene and induce cell cytotoxicity in the presence of G-418. In JFH-1-infected cells, moreover, the attachment of mPUM to the IRES may also lead to the reduction in viral polyprotein translation and subsequent decrease in the production of viral structural and nonstructural proteins. Since nonstructural proteins comprise the main components of HCV replicase complex, their decline can lead to the reduction of HCV RNA replication [12–14].

Stem-loop III involves in the HCV replication not only by affecting HCV translation but also by enhancing RNA replication efficiency [6,15,16]. Addition of stem-loop III to stem-loops I and II, which are sufficient for HCV replication, augmented RNA replication to about 100-fold. Therefore, the binding of mPUM protein to stem-loop IIIb may also affect HCV replication through reduction of the efficiency of RNA replication.

HCV RNA replication involves production of replicative intermediate minus sense (-) RNA from positive sense (+) genomic RNA by viral RNA-dependent RNA polymerase and subsequent transcriptions of progeny genomic (+) RNAs from (-) RNA by the same enzyme. Several studies have demonstrated that a replication region (the first 157-nt of HCV genome, including stem-loops I and II) is necessary and sufficient for RNA replication [6,17], because the introduction of any mutation or deletion to the 3′ terminal sequences of (-) RNA reduced or completely blocked replication [6,18]. Binding of mPUM protein to the stem-loop IIIb may sterically hinder the activity of RNA-dependent RNA polymerase for transcription of the 3′ terminal sequences of the viral (-) RNA (the replication region, upstream of subdomain IIIb) from the viral (+) RNA. Thus, the replication signals that function as promoters for initiation of (+) RNA transcription may not be available in the 3′ end of HCV (-) RNA [7,18,19].

On the other hand, the binding of mPUM protein may alter the secondary structure of downstream and/or upstream sequences flanking its binding site. Thus, some cellular factors such as polypyrimidine tract-binding protein, poly(C)-binding protein 2, La autoantigen and synaptotagmin-binding, cytoplasmic RNA-interacting protein, which bind to *cis*-elements within or out of replication region and miR-122 that interacts directly with two sites (nucleotides 22–29 and 38–43) in the replication region, may not be able to interact with their specific sites in the 5′-UTR [15–17,20,21]. Since current data indicate that these factors are necessary for efficient replication, any change in the secondary structure at their binding sites may lead these factors not to positively regulate RNA replication.

Our results (Figure 2A) suggest that the expression of mPUM protein for 48 h before infection of the Huh7.5 cells was able to significantly induce a more than 98% reduction in HCV RNA copy numbers, 9 days after infection. These findings are also in line with the results of the cytotoxicity assessment of the mPUM expression in JFH-1-infected Huh7.5 cells that showed a significant reduction in the level of cell cytotoxicity compared with that of naive Huh7.5 cells, 9 days postinfection. Additional experiments are required to have a better view on the timing of mPUM action; however, since the cell culture-adapted strain of HCV needs at least 6 days to complete a replication cycle, this may explain the reason why it takes 9 days postinfection to observe the potential antiviral effects of the mPUM.

Comparison of cell cytotoxicity between infected and naive cells on days 3, 6 and 9 demonstrated that HCV infection does not cause any significant CPE until day 3 and CPEs induced by the infection was started from day 6 and continued to day 9. This was in accordance with the results obtained by Ferraris *et al.* [10] who showed that CPEs of an optimized JFH-1 virus started 6 days postinfection. Our results also indicated that Huh7.5 cells expressing mPUM or wtPUM had similar viability after HCV challenge. These preliminary findings reveal that the observed inhibition of HCV replication is not due to the decreased cell viability. The obvious lack of cytotoxicity is further supported by the observation that Huh7.5 cells could be propagated during 11 days despite expressing functional mPUM protein. Together these findings suggest that the expression of mPUM is not cytotoxic, even at levels enough to reduce HCV translation and replication.

In this study using the approach of modified RBPs provided the evidence for the repression of HCV RNA replication. To the best of our knowledge, this is the first study to target a viral RNA using an engineered sequence-specific RBP. However, other strategies targeting the 5′-UTR of HCV genome, using antisense oligonucleotides have also been described previously [22–24]. For example, siRNAs, DNAzymes and ribozymes have been designed to bind to 5′-UTR in order to trigger the cleavage of HCV IRES and subsequently to repress viral replication in cell culture. In agreement with our data, Korf *et al.* [22] demonstrated approximately 60% reduction in HCV replication, using siRNAs directed against stem-loops II and III. Moreover, Roy *et al.* [23] designed several DNAzymes targeting stem-loop IIIc with an inhibitory activity of approximately 70% reduction in replicon RNA synthesis. In another study, Chevalier *et al.* [24] showed a greater than 96% reduction in focus-forming units/ml infectious titer, using a siRNA targeting both 3′ end of domain III and 5′ end of domain IV involved in a pseudoknot-located upstream from the initiator codon. In our study, transfection of mPUM 48 h before infection with JFH-1 strain induced a greater than 98% reduction in viral RNA copy numbers/ml in the supernatant of Huh-7.5 cells, 9 days after infection.

The present study had some limitations. Although our results showed significant reduction of viral RNA copy number/ml, the assessment of virus infectivity through performing a plaque assay would be ideal to provide stronger support for the action of the modified protein to prevent HCV replication. Performing the reporter assay with another viral IRES could also provide us with stronger evidence to confirm sequence-specific behavior of the mPUM protein.

The approach used in this study is probably not directly useful in the case of HCV for which there are effective and safe direct-acting antivirals. The direct-acting antivirals are considered the ideal choice for the treatment of HCV infection due to their high efficacy (sustained virologic response >90%) and minimal adverse effects. However, this strategy can set the stage for using modified PUM-HDs as an RNA-binding protein for interfering with critical steps such as replication and translation in viral life cycle, especially in the case of RNA viruses for which there are no effective antivirals.

## Conclusion

Recruitment of a natural host protein that is modified to sequence-specifically bind to vital segments of a virus genome can provide us with a new flexible toolkit to repress the replication of different viruses. These proteins not only can be programmed to bind different segments of the genome of RNA viruses but also they can be targeted for binding to mRNA species of essential viral proteins in the replication cycle of DNA viruses.

**Summary points**

- A sequence-specific RNA-binding protein was modified for binding to the internal ribosome entry site (IRES) segment of hepatitis C virus (HCV) genome.
- Modified protein reduced IRES-dependent luciferase reporter gene translation more than 30%.
- Modified protein reduced IRES-dependent *neo*-resistance gene translation and rendered Huh7.5 clone 5 cells sensitive to G-418 more than 70%.
- Modified protein reduced cell cytotoxicity of HCV-infected cells to the level of uninfected cells.
- Modified protein reduced HCV viral load more than 98%.
- PUF proteins are new flexible toolkits to target the replication of viruses, especially RNA viruses.
